# Type 2 diabetes and risk of colorectal cancer in two large U.S. prospective cohorts

**DOI:** 10.1038/s41416-018-0314-4

**Published:** 2018-11-07

**Authors:** Yanan Ma, Wanshui Yang, Mingyang Song, Stephanie A. Smith-Warner, Juhong Yang, Yanping Li, Wenjie Ma, Yang Hu, Shuji Ogino, Frank B. Hu, Deliang Wen, Andrew T. Chan, Edward L. Giovannucci, Xuehong Zhang

**Affiliations:** 1 School of Public Health, China Medical University, Shenyang, Liaoning P.R. China; 2 Channing Division of Network Medicine, Department of Medicine, Brigham and Women’s Hospital and Harvard Medical School, Boston, MA USA; 3 Department of Nutrition, School of Public Health, Anhui Medical University, Hefei, Anhui P.R. China; 4 Department of Nutrition, Harvard T.H. Chan School of Public Health, Boston, MA USA; 5 Clinical and Translational Epidemiology Unit, Massachusetts General Hospital and Harvard Medical School, Boston, MA USA; 6 Division of Gastroenterology, Massachusetts General Hospital, Boston, MA USA; 7 Department of Epidemiology, Harvard T.H. Chan School of Public Health, Boston, MA USA; 8 2011 Collaborative Innovation Center of Tianjin for Medical Epigenetics, Key Laboratory of Hormone and Development (Ministry of Health), Metabolic Disease Hospital and Tianjin Institute of Endocrinology, Tianjin Medical University, 300070 Tianjin, P.R. China; 9 Department of Epidemiology, Harvard T.H. Chan School of Public Health, Boston, MA USA; 10 Department of Oncologic Pathology, Dana-Farber Cancer Institute and Harvard Medical School, Boston, MA USA; 11 Broad Institute of Massachusetts Institute of Technology and Harvard, Cambridge, MA USA; 12 Program in MPE Molecular Pathological Epidemiology, Department of Pathology, Brigham and Women’s Hospital and Harvard Medical School, Boston, MA USA; 13 Department of Immunology and Infectious Diseases, Harvard T.H. Chan School of Public Health, Boston, MA USA

## Abstract

**Background:**

Previous studies have shown a positive association between type 2 diabetes (T2D) and colorectal cancer (CRC) risk. However, it is uncertain whether this association differs by duration of T2D or sex. We thus investigated the associations of T2D and its duration with the risk of incident CRC.

**Methods:**

We followed 87,523 women from the Nurses’ Health Study (1980–2012) and 47,240 men from the Health Professionals Follow-up Study (1986–2012). Data on physician-diagnosed T2D was collected at baseline with a questionnaire and updated biennially. Cox regression models were used to estimate multivariable-adjusted hazard ratios (HRs) and 95% confidence intervals (CIs).

**Results:**

We documented 3000 CRC cases during up to 32 years of follow-up. Among men, T2D was associated with increased risk of CRC compared to those without T2D (HR: 1.42; 95% CI: 1.12–1.81). This positive association persisted in sensitivity analyses by excluding CRC identified within 1 year of diabetes diagnosis and patients with T2D who used hypoglycaemic medications. Among women, T2D was positively, but not statistically significantly, associated with CRC risk (HR: 1.17; 95% CI: 0.98–1.39).

**Conclusions:**

Our findings support that T2D was associated with a moderately higher risk of developing CRC in men; a weaker, nonsignificant positive association was observed in women.

## Introduction

Colorectal cancer is the third most commonly diagnosed cancer in the US^1^ and worldwide.^2^ A number of epidemiological studies have shown that individuals with type 2 diabetes have a higher risk of developing colorectal cancer compared with their non-diabetic counterparts.^3–22^ Elevated insulin due to insulin resistance and hyperinsulinaemia, which occurs in the early stages of type 2 diabetes,^23^ has been hypothesised to partially explain the proposed type 2 diabetes–colorectal cancer association.^24^ Insulin is an important growth factor of colonic epithelial cells and is a mitogen of tumour cell growth in vitro.^24^ Insulin may also promote colorectal carcinogenesis indirectly through increasing bioavailable insulin-like growth factor 1, which could enhance cell proliferation and inhibit apoptosis.^24^ Another potential biological link between type 2 diabetes and cancer is hyperglycaemia, which may impair the effect of ascorbic acid on intracellular metabolism, reduce the effectiveness of the immune system, and regulate the level of reactive oxygen species, which could favour cancer development and progression in diabetic patients.^25^

Several issues regarding the association between type 2 diabetes and colorectal cancer risk remained unresolved. First, the relationship between duration of diabetes and colorectal cancer risk remains uncertain. Some studies have reported a higher risk of colorectal cancer with prolonged duration of type 2 diabetes^3,5,26^ or with short duration,^13^ whereas other studies have shown no difference in risk by duration of type 2 diabetes.^7,12^ Second, most previous studies only examined a single measurement of diabetes at baseline,^4,6,8,10,14,15^ which cannot account for new diagnoses of diabetes during follow-up. Last, published data has shown inconclusive results about the association of type 2 diabetes with the risk of different subsites of colorectal cancer.^27–29^ The potential gender difference in the type 2 diabetes and colorectal cancer association also remains unclear. This issue is of interest because sex hormones (e.g. testosterone, oestrogen and progesterone) may play a role in colorectal cancer.^30–35^

To address these issues, we examined whether physician-diagnosed type 2 diabetes and its duration are associated with the risk of colorectal cancer in the Nurses’ Health Study and the Health Professionals Follow-up Study. In addition, we also examined potential differences in the association by sex.

## Methods

### Study population

We conducted this study by using data from the two large-scale prospective cohort studies: the Nurses’ Health Study and the Health Professionals Follow-up Study. In brief, the Nurses’ Health Study was initiated in 1976 and included a total of 121,700 US female registered nurses aged 30–55 years. The Health Professionals Follow-up Study was established in 1986 and included 51,529 male health professionals aged 40–75 years. Participants were followed up biennially by validated questionnaires on demographics, medical conditions, and health-related behaviours. The validity of questionnaire has been described in detail previously.^36^ The follow-up rates have been >90% in each cohort.

For the current study, we used 1980 as the baseline for the Nurses’ Health Study and 1986 for the Health Professionals Follow-up Study. We excluded participants with missing data on diabetes, with unconfirmed diabetes, and with a history of cancer (except for non-melanoma skin cancer) or inflammatory bowel disease at baseline. After exclusions, the final study population consisted of 87,523 women from the Nurses’ Health Study and 47,240 men from the Health Professionals Follow-up Study. The study protocol was approved by the institutional review board of the Brigham and Women’s Hospital and the Human Subjects Committee Review Board of the Harvard T. H. Chan School of Public Health.

### Assessment of type 2 diabetes

In the baseline and all biennial questionnaires, the participants were asked if and when they had ever been diagnosed with type 2 diabetes by a physician. To confirm the self-reported cases of physician-diagnosed diabetes, a supplementary questionnaire was then sent for ascertainment to obtain details about the date of diagnosis, symptoms, diagnostic tests, and hypoglycaemic treatment. Beginning in 1986 in each cohort, supplementary questionnaires were administered to collect information on antidiabetic drug use (insulin or hypoglycaemic agent) as described in detail previously.^37^ Cases of type 2 diabetes were defined as initially self-reported diabetes and subsequently confirmed by the validated questionnaires.^38,39^ Consistent with previous studies from the same cohorts,^40^ confirmed diabetes requires at least one of the following criteria reported on the supplementary questionnaire according to the American Diabetes Association: one or more classic symptoms (excessive thirst, polyuria or frequent urination, weight loss, hunger) plus fasting plasma glucose ≥7.0 mmol/l or random plasma glucose levels ≥11.1 mmol/l; ≥2 elevated plasma glucose concentrations on different occasions (fasting glucose ≥7.0 mmol/l, random plasma glucose ≥11.1 mmol/l, and/or plasma glucose ≥11.1 mmol/l after ≥2 h shown by oral glucose tolerance testing) in the absence of symptoms; or treatment with hypoglycaemic medication. Before 1998, fasting plasma glucose of 7.8 mmol/l was considered for the diagnosis of diabetes according to the National Diabetes Data Group criteria. The validity of the supplementary questionnaire has been confirmed through medical records. In each cohort, diagnosis of type 2 diabetes was confirmed in >97% of the cases.^39,41^

### Assessment of covariates

Information on body weight, physical activity, other lifestyle information, and medical history was obtained at baseline and was updated through biennial questionnaires. Height was ascertained in 1980 in the Nurses’ Health Study and in 1986 in the Health Professionals Follow-up Study. We calculated body mass index (BMI) as weight in kilograms divided by height in metres squared. Early-life BMI was measured at 18 years in the Nurses’ Health Study and at 21 years in the Health Professionals Follow-up Study. Each cohort administered food-frequency questionnaires (~130 food items) to obtain information on usual dietary intake over the previous year at baseline and repeated almost every 4 years thereafter, which allowed us to calculate intakes of nutrients and foods such as alcohol, calories, calcium, dietary fibre, sugar, carbohydrates, folate, vitamin D, processed meat, and red meat.^42–46^

### Identification of colorectal cancer cases

In both cohorts, we identified cases of colorectal cancer through biennial questionnaires.^47^ All possible cancer cases were confirmed through review of medical and pathological records. The incident colorectal cancer cases were defined as a primary tumour with International Classification of Diseases-9 codes of 153 and 154. A study physician who was blinded to exposure data abstracted information on tumour anatomic location, stage, and histology. We searched state vital statistics records and the National Death Index to identify additional unreported cancer deaths. In this analysis, we considered both overall colorectal cancer and anatomic subsites, including colon cancer, proximal colon cancer (caecum, ascending colon, and transverse colon), distal colon cancer (descending colon and sigmoid colon), and rectal cancer (including recto-sigmoid).

### Statistical analyses

Person-time for participants was calculated from the return of the baseline questionnaires until the date of colorectal cancer diagnosis, death, loss to follow-up, or the end of the follow-up (June 2012 for the Nurses’ Health Study and January 2012 for the Health Professionals Follow-up Study), whichever came first. Consistent with our previous report,^7^ we evaluated the association with status (no versus yes) and duration of type 2 diabetes (0.1–5, 5.1–10, 10.1–15, and >15 years) within each cohort. We used Cox regression models to calculate the multivariable hazard ratios (HRs) and 95% confidence intervals (95% CIs). Age in months and calendar year at the start of follow-up of each 2-year questionnaire cycle were used as stratification variables in the model. In the multivariable analyses, we adjusted for race (Whites or non-Whites); family history of diabetes (yes or no); regular aspirin use (yes or no); BMI (<25, 25–<27.5, 27.5–<30, ≥30 kg/m^2^); history of colorectal cancer in a parent or sibling (yes or no); history of endoscopy/sigmoidoscopy (yes or no); smoking habits (0, 1–10, or >10 pack-years); alcohol consumption (<5, 5–<15, ≥15 g/day); physical activity (<3, 3–<27, ≥27 METS-hours/week); total calories (tertiles); energy-adjusted intake of total folate; total calcium; total vitamin D; processed meat; and beef, pork, or lamb as a main dish (all in tertiles). For women, we additionally adjusted for postmenopausal hormone use (premenopausal, never, past, or current user). Confounders were updated over the follow-up periods to reduce random within-person measurement error. Test of statistical heterogeneity in the association of type 2 diabetes and colorectal cancer risk by gender was assessed by the Q statistics.^48^ For the two cohorts, there were about 0.1–3% missing data in processed meat, beef, pork, or lamb as the main dish or for family history of colorectal cancer. Given the generally low proportion of missing data in these covariates, we have included the individuals with missing in family history of colorectal cancer in the no family history group and missing in intake of processed meat or beef, pork, or lamb as the main dish in the tertile 1 group.

Previous studies have suggested that smoking^20^ may modify the association between diabetes and colorectal cancer; and BMI, physical activity, and moderate alcohol consumption are associated with insulin resistance.^49–51^ Therefore, we considered these potential variables that might modify the diabetes and colorectal cancer risk associations. We conducted subgroup analyses by smoking status (never smoker versus ever smoker), BMI (≥25 versus <25 kg/m^2^), early life BMI (≥23 versus <23 kg/m^2^), physical activity (<9 versus ≥9 METS-hours/week), alcohol drinking (non-drinkers versus drinkers), and other selected risk factors including age (≥65 versus <65 years) and family history of diabetes (yes versus no). We used Wald test to examine whether the cross-product terms between these variables and diabetes status were statistically significant.

We conducted several secondary analyses. First, we evaluated whether the associations between diabetes (status and duration) and colorectal cancer risk differed by anatomic subsites (colon cancer, proximal colon cancer, distal colon cancer, and rectal cancer).^28^ Second, we restricted analyses to untreated diabetes by excluding patients with type 2 diabetes who have used hypoglycaemic medication (insulin or oral hypoglycaemic agent). A total of 195 cases (146 for women and 49 for men) were excluded from our analysis to investigate whether diabetes treatments affect the association between type 2 diabetes and colorectal cancer risk. Last, we excluded colorectal cancer cases diagnosed within 1 year of diabetes diagnosis to minimise the potential for reverse causality and detection bias.

All statistical analyses were conducted using the SAS software (Version 9.3; SAS Institute, Cary, NC, USA). All statistical tests were two sided, and the significance level was set at *P* < 0.05.

## Results

During 3,547,837 person-years of follow-up, we documented a total of 3000 incident cases of colorectal cancer (1790 in women and 1210 in men). In both cohorts, participants with type 2 diabetes were older and more likely to smoke and have a family history of diabetes (Table 1). Moreover, participants with type 2 diabetes were more likely to have higher BMI and use aspirin and were less likely to be physically active, drink alcohol, and consume sugar. Patients with longer duration of type 2 diabetes generally tended to be more physically active and consume less sugar and carbohydrates but were more likely to consume processed meats (especially in men).

**Table 1:** Age-standardised characteristics of participants according to status and duration of type 2 diabetes in the Nurses’ Health Study (NHS) and Health Professionals Follow-up Study (HPFS)^a^

	No diabetes	Diabetes	0.1–5 years	5.1–10 years	10.1–15 years	>15 years
Women (NHS)						
Age (year)^b^	60.2 (11.3)	67.7 (9.4)	65.6 (9.3)	67.5 (9.3)	68.5 (9.4)	70.0 (9.2)
Duration of diabetes, years	NA	9.4 (8.2)	2.4 (1.5)	7.4 (1.4)	12.3 (1.4)	22.0 (6.4)
Whites, %	97.6	95.6	95.9	95.4	95.6	95.7
Body mass index, kg/m^2^	24.7 (4.2)	30.2 (5.7)	30.1 (5.5)	30.4 (5.7)	30.4 (5.9)	29.4 (5.9)
BMI at age 18 years, kg/m^2^	21.2 (2.9)	22.9 (4.0)	22.5 (3.9)	22.8 (4.0)	23.0 (4.1)	23.2 (4.2)
Physical activity, METS-hours/week	16.9 (17.6)	12.7 (12.9)	13.0 (13.6)	12.7 (12.9)	12.7 (12.5)	12.6 (12.2)
Smoking, pack years	12.9 (19.0)	13.8 (20.0)	14.2 (20.2)	13.9 (20.1)	13.8 (20.2)	12.9 (19.8)
Regular aspirin use, %	39.9	47.3	44.9	46.2	49.3	50.9
History of endoscopy/sigmoidoscopy, %	26.1	26.6	27.4	26.8	25.4	26.5
Family history of colorectal cancer, %	12.2	12.6	12.6	12.8	12.4	12.9
Family history of diabetes, %	26.6	55.9	50.7	54.8	58.5	63.7
Postmenopausal status, %	81.7	85.3	84.5	85.0	86.1	87.0
Postmenopausal hormone use, %	48.1	45.4	46.7	44.4	45.2	46.6
Total calorie intake, kcal/day	1674 (443)	1707 (452)	1719 (451)	1701 (455)	1697 (453)	1705 (448)
Alcohol, g/day	6.2 (9.4)	3.0 (6.7)	3.4 (6.9)	2.9 (6.7)	2.6 (6.4)	2.8 (7.1)
Total calcium, g/day	933 (359)	912 (338)	911 (340)	910 (339)	907 (335)	929 (340)
Total fibre, g/day	17.5 (4.9)	17.6 (4.7)	17.3 (4.6)	17.5 (4.7)	17.7 (4.6)	18.0 (4.7)
Total sugar, g/day	94.3 (26.7)	90.5 (26.1)	93.0 (26.4)	90.4 (25.8)	88.0 (25.5)	88.1 (26.0)
Total carbohydrates, g/day	181 (32)	179 (30)	181 (30)	179 (31)	178 (30)	178 (30)
Total folate, mcg/day	424 (213)	424 (198)	417 (199)	423 (201)	424 (191)	442 (195)
Vitamin D intake (IU/day)	356 (224)	358 (210)	351 (212)	358 (214)	360 (205)	376 (204)
Processed meat, servings/week	0.94 (1.26)	1.19 (1.45)	1.15 (1.44)	1.20 (1.47)	1.23 (1.48)	1.19 (1.38)
Beef/pork/lamb as the main dish, servings/week	2.07 (1.43)	2.24 (1.46)	2.21 (1.43)	2.27 (1.52)	2.28 (1.47)	2.24 (1.42)
Men (HPFS)						
Age (year)^b^	63.8 (11.2)	70.7 (9.1)	68.2 (9.1)	71.4 (8.7)	73.4 (8.2)	76.2 (8.0)
Duration of diabetes, years	NA	5.7 (4.8)	2.3 (1.5)	7.2 (1.4)	12.1 (1.3)	17.8 (2.2)
Whites, %	96.0	92.3	93.0	92.4	91.6	92.1
Body mass index, kg/m^2^	25.7 (3.3)	29.1 (4.6)	28.8 (4.4)	29.1 (4.5)	29.5 (4.4)	28.4 (4.8)
BMI at age 21 years, kg/m^2^	22.0 (5.5)	23.4 (5.8)	23.1 (5.9)	23.4 (5.7)	23.9 (5.1)	23.3 (5.0)
Physical activity, METS-hours/week	29.7 (28.9)	24.2 (23.8)	24.0 (23.7)	24.6 (22.9)	24.9 (22.0)	30.5 (23.5)
Smoking, pack years	11.8 (18.1)	13.7 (19.1)	14.3 (19.7)	13.5 (18.7)	12.5 (16.9)	9.7 (15.1)
Regular aspirin use, %	38.1	46.1	47.2	47.4	45.8	39.5
History of endoscopy/sigmoidoscopy, %	53.5	60.7	59.0	62.5	68.8	61.6
Family history of colorectal cancer, %	12.2	11.4	11.5	11.5	11.9	10.7
Family history of diabetes, %	25.0	47.5	46.9	46.8	47.6	38.1
Total calorie intake, kcal/day	1977 (555)	1991 (564)	1995 (564)	1992 (544)	1965 (503)	1815 (526)
Alcohol, g/day	11.1 (13.9)	8.5 (12.2)	8.8 (12.4)	8.6 (12.3)	7.8 (10.8)	12.1 (13.7)
Total calcium, g/day	935 (373)	936 (354)	930 (357)	949 (344)	959 (324)	927 (362)
Total fibre, g/day	22.1 (6.6)	21.9 (6.0)	21.6 (6.0)	22.2 (5.9)	22.6 (5.8)	21.5 (6.4)
Total sugar, g/day	110.5 (30.4)	104.1 (27.3)	106.5 (27.7)	102.8 (26.2)	99.6 (23.4)	89.3 (29.4)
Total carbohydrates, g/day	243 (39)	237 (34)	238 (35)	237 (33)	236 (32)	226 (36)
Total folate, mcg/day	542 (254)	545 (231)	534 (232)	558 (222)	579 (208)	572 (218)
Vitamin D intake (IU/day)	434 (267)	424 (243)	422 (247)	431 (233)	430 (212)	463 (201)
Processed meat, servings/week	1.08 (1.49)	1.31 (1.45)	1.27 (1.45)	1.33 (1.54)	1.33 (1.31)	1.54 (1.30)
Beef/pork/lamb as the main dish, servings/week	1.72 (1.40)	1.96 (1.42)	1.95 (1.44)	1.94 (1.29)	1.94 (1.25)	2.05 (1.15)

Values were means (SD) or percentages and were standardised to the age distribution of the study population

Values of polytomous variables may not sum to 100% due to rounding

^a^Updated information over the study follow-up was used

^b^Value was not age adjusted

Among men, type 2 diabetes was associated with increased risk of incident colorectal cancer (multivariable HR: 1.42; 95% CI: 1.12–1.81) (Table 2). The positive association for men persisted in sensitivity analyses excluding colorectal cancer cases diagnosed within 1 year of type 2 diabetes diagnosis (multivariable HR: 1.40; 95% CI: 1.10–1.78). Additionally, we observed similar positive associations for each tumour anatomic subsite (Supplementary Table 1) and when we restricted cases to only those with untreated diabetes (Supplementary Table 2*)*. Among women, type 2 diabetes showed only a modest, borderline significant association with colorectal cancer risk (HR: 1.17; 95% CI: 0.98–1.39) (Table 2). The heterogeneity test did not achieve statistical significance (*P* = 0.20 for men versus women). Colorectal cancer risk did not vary with type 2 diabetes duration in either men or women.

**Table 2:** Status and duration of type 2 diabetes and colorectal cancer risk in the Nurses’ Health Study (NHS) and Health Professionals Follow-up Study (HPFS)

	Women (NHS, *N* = 1790)		Men (HPFS, *N* = 1210)	
	No. of cases	HR (95% CI)	No. of cases	HR (95% CI)
No diabetes	1631	1 (Reference)	1129	1 (Reference)
Diabetes				
Age-adjusted model	159	**1.32 (1.12, 1.55)**	81	**1.57 (1.24, 1.98)**
Multivariable-adjusted model^a^	159	1.17 (0.98, 1.39)	81	**1.42 (1.12, 1.81)**
0.1–5 years				
Age-adjusted model	51	1.32 (1.00, 1.75)	37	**1.51 (1.08, 2.12)**
Multivariable-adjusted model^a^	51	1.16 (0.87, 1.54)	37	1.39 (0.99, 1.95)
5.1–10 years				
Age-adjusted model	42	1.38 (1.01, 1.88)	25	**1.59 (1.06, 2.38)**
Multivariable-adjusted model^a^	42	1.20 (0.88, 1.64)	25	1.42 (0.94, 2.15)
10.1–15 years				
Age-adjusted model	24	1.15 (0.77, 1.72)	14	1.71 (1.00, 2.92)
Multivariable-adjusted model^a^	24	1.01 (0.67, 1.52)	14	1.53 (0.89, 2.64)
>15 years				
Age-adjusted model	42	**1.37 (1.00, 1.87)**	5	1.59 (0.65, 3.89)
Multivariable-adjusted model^a^	42	1.26 (0.91, 1.72)	5	1.37 (0.56, 3.38)

^a^Adjusted for age; race (Whites or non-Whites); family history of diabetes (yes or no); regular aspirin use (yes or no); BMI (<25, 25–<27.5, 27.5–<30, ≥30 kg/m^2^); history of colorectal cancer in a parent or sibling (yes or no); history of endoscopy/sigmoidoscopy (yes or no); smoking (0, 0–<10, ≥10 pack-years); alcohol consumption (<5, 5–<15, ≥15 g/day); physical activity (<3, 3–<27, ≥27 METS-hours/week); total calorie intake (tertiles); energy-adjusted intake of total folate; total calcium; total vitamin D intake; processed meats; and beef, pork, or lamb as a main dish (all in tertiles). We also adjusted for postmenopausal hormone use (premenopausal, never, past, or current user) in women. Bold text indicated a statistically significant association

In stratified analyses (Table 3), the association between type 2 diabetes and colorectal cancer risk appeared slightly stronger among ever smokers (*P-interaction* = 0.04 for women) and non-drinkers (*P-interaction* = 0.05 for men). Additionally, the association was also slightly stronger for those with higher BMI or early life BMI and individuals with a family history of diabetes, but none of the *P*-values were statistically significant.

**Table 3:** Stratified analyses of type 2 diabetes and risk of colorectal cancer in the Nurses’ Health Study (NHS) and Health Professionals Follow-up Study (HPFS)^a^

	Women (NHS, *N* = 1790)		Men (HPFS, *N* = 1210)	
	No. of cases/person-years	HR (95% CI)	No. of cases/person-years	HR (95% CI)
Age				
<65 years	795/1,645,237	1.10 (0.79, 1.54)	349/553,483	1.73 (0.98, 3.04)
≥65 years	995/883,406	1.20 (0.97, 1.47)	861/465,709	**1.37 (1.05, 1.79)**
* P-interaction*	0.58		0.26	
BMI				
<25 kg/m^2^	926/1,510,733	0.97 (0.61, 1.55)	456/456,193	1.28 (0.73, 2.24)
≥25 kg/m^2^	864/1,017,911	**1.24 (1.03, 1.50)**	754/563,000	**1.52 (1.16, 1.99)**
* P-interaction*	0.22		0.78	
Early life BMI^b^				
** <**23 kg/m^2^	1342/1,997,616	1.16 (0.93, 1.45)	639/536,124	**1.44 (1.02, 2.03)**
** ≥**23 kg/m^2^	448/531,028	**1.40 (1.06, 1.85)**	571/483,068	**1.50 (1.07, 2.10)**
* P-interaction*	0.19		0.74	
Smoking				
Never	780/1,180,925	0.95 (0.72, 1.27)	546/532,379	1.35 (0.93, 1.97)
Ever	1010/1,347,719	**1.37 (1.09, 1.72)**	664/486,814	**1.57 (1.15, 2.16)**
* P-interaction*	**0.04**		0.95	
Alcohol consumption^c^				
Non-drinkers	366/564,745	1.37 (0.99, 1.89)	199/180,951	**2.00 (1.14, 3.52)**
Drinkers	1424/1,963,899	1.10 (0.89, 1.36)	1011/838,241	1.22 (0.93, 1.60)
* P-interaction*	0.54		0.05	
Physical activity				
<9 METS-hours/week	893/1,396,849	1.16 (0.90, 1.48)	303/230,259	**1.70 (1.05, 2.75)**
≥9METS-hours/week	897/1,131,795	1.16 (0.90, 1.50)	907/788,934	**1.37 (1.03, 1.82)**
* P-interaction*	0.68		0.35	
Family history of diabetes				
No	1251/1,817,196	1.09 (0.84, 1.41)	880/754,867	1.39 (1.00, 1.95)
Yes	539/711,448	1.25 (0.98, 1.60)	330/264,325	**1.48 (1.03, 2.13)**
* P-interaction*	0.38		0.70	

^a^Adjusted for age; race (Whites or non-Whites); family history of diabetes (yes or no); regular aspirin use (yes or no); BMI (<25, 25–<27.5, 27.5–<30, ≥30 kg/m^2^); history of colorectal cancer in a parent or sibling (yes or no); history of endoscopy/sigmoidoscopy (yes or no); smoking (0, 0–<10, ≥10 pack-years); alcohol consumption (<5, 5–<15, ≥15 g/day); physical activity (<3, 3–<27, ≥27 METS-hours/week); total calorie intake (tertiles); energy-adjusted intake of total folate; total calcium; total vitamin D intake; processed meats; and beef, pork, or lamb as a main dish (all in tertiles). Women were also adjusted for postmenopausal hormone use (premenopausal, never, past, or current user). Of note, variables examined in this Table were not adjusted for

^b^BMI was measured at 18 and 21 years for women and men, respectively

^c^Alcohol consumption (0, 0–<20, ≥20 g/day) for women and (0, 0–<30, ≥30 g/day) for men.

Bold text indicated a statistically significant association

## Discussion

In these two large prospective cohort studies, with >26 years of follow-up, we found that type 2 diabetes was associated with a moderately higher risk of incident colorectal cancer in men; a weaker, nonsignificant positive association was observed in women. The positive association for men remained in sensitivity analyses that accounted for potential reverse causality by excluding colorectal cancer cases diagnosed within 1 year of diagnosis of type 2 diabetes and diabetes treatments and did not vary by diabetes duration.

In our previous study with follow-up through 1994,^7^ we reported a significant association between type 2 diabetes and colorectal cancer risk among women in the Nurses’ Health Study (HR: 1.43; 95% CI: 1.10–1.87). Positive associations of similar magnitude were reported for the women in the Cancer Prevention Study II^17^ and the Iowa Women’s Health Study^16^ with follow-up through 1998 and 1999, respectively. In 2005, a meta-analysis of 15 studies (6 case–control and 9 cohort studies, including our prior Nurses’ Health Study report) reported positive associations for diabetes and colorectal cancer risk of similar magnitude for men (HR: 1.29; 95% CI: 1.15–1.44) and women (HR: 1.33; 95% CI: 1.23–1.44).^9^ However, most subsequent studies have reported a weaker positive^14^ or a null association^3,18,20–22^ between type 2 diabetes and colorectal cancer risk among women. This was consistent with our observation of a slightly weaker association in women but a moderately higher-risk association in men.

The possible differential association between women and men observed might be due to potential sex differences in the use of metformin and degree of glucose control.^3^ Compared with men, metformin has been more commonly used among women since the late 1990s.^52^ However, the potential effect of metformin use and colorectal cancer risk is not completely understood and more better designed studies are warranted to quantify the magnitude of the association between metformin use and colorectal cancer risk. Additionally, women generally tend to have better glucose control than men, even among those with type 2 diabetes.^3^ In fact, among individuals with type 2 diabetes, this difference is increasing. The National Health and Nutrition Examination Survey data showed that women with type 2 diabetes, but not men, have statistically significantly improved haemoglobin A1c (an indicator of glucose control) during the period from 1999 to 2004 in the US.^53^ Furthermore, differences in sex hormones may contribute to a more insulin-sensitive environment in women than in men. It may protect women from adverse outcomes related to hyperinsulinaemia.^54,55^ Observational studies^30–34,56^ suggested an inverse association of endogenous oestrogens with the risk of colorectal cancer among women. Particularly, compared with women, men may have a higher insulin resistance partly due to the lack the protective effect of oestrogen.^54,55^ Despite relatively comprehensive research on type 2 diabetes in relation to risk of colorectal cancer,^29^ current findings are inconsistent with regard to the potential gender difference. We observed a slightly stronger association in men than in women in this study but there was no statistically significant heterogeneity by gender. Nonetheless, the association between type 2 diabetes and colorectal cancer is strongly influenced by shared risk factors such as genetic variants, obesity, smoking, alcohol, and lack of physical activity.^57,58^ These potential factors might modify the diabetes and colorectal cancer risk differently in women and men, which requires further investigation.

We found that the type 2 diabetes–colorectal cancer association did not appear to be associated with diabetes duration. Because beta cells in the pancreas produce lower insulin levels as diabetes progresses into later stage, if hyperinsulinaemia plays a key role in this association, stronger positive associations would be expected in the early stage of diabetes. Thus our results did not support a strong role of hyperinsulinaemia in diabetes–colorectal cancer association.

We found slightly stronger associations between type 2 diabetes and colorectal cancer among ever smokers. This observation was consistent with a population-based, retrospective cohort study, which reported a stronger positive association among smokers.^20^ We also found a stronger association among non-drinkers of alcohol, indicating a potential interactive effect of alcohol consumption on type 2 diabetes. A similar, though not statistically significant, pattern was observed in women. Similar to that seen in cardiovascular disease,^59^ light-to-moderate intake of alcohol was associated with the reduced risk of colorectal cancer in some studies.^60–62^ Light-to-moderate alcohol consumption may increase insulin sensitivity and thus reduce insulin production.^49,50^ This was consistent with the observation that moderate alcohol consumption was associated with lower levels of plasma C-peptide and lower risk of colorectal cancer.^63^ Thus the potential interaction between insulin and alcohol may protect patients with type 2 diabetes from serious outcomes of hyperinsulinaemia. However, the underlying mechanisms warrant further investigation.

Our present study has several strengths, including the prospective cohort design, high follow-up rates, validated colorectal cancer outcomes, and the use of repeated measures of physician-confirmed diabetes and lifestyle data. Our study also has several limitations. First, we have limited data on diabetes treatment. The observed association could be influenced by diabetes treatments because medications used to treat diabetes such as insulin injection^64^ or metformin use^65^ may affect the incidence of colorectal cancer. Moreover, we have limited data on glucose control because hyperglycaemia will also affect the cancer risk. However, the results were similar when we restricted the analysis among untreated diabetes (without any use of insulin or other hypoglycaemic agents). Second, our results might be influenced by detection bias, because newly diagnosed diabetes is more likely to be diagnosed with cancer due to increased detection around the time of the diagnosis of diabetes.^66^ However, we observed similar positive association when we excluded cases diagnosed within 1 year since type 2 diabetes.

In summary, our study suggests that type 2 diabetes was associated with a moderately higher risk of incident colorectal cancer in men; a weaker, nonsignificant association was observed in women. More studies are warranted to further investigate the underlying mechanisms. Our finding further underscores the importance of enhancing the effort to reduce the emerging epidemics of type 2 diabetes for colorectal cancer prevention and control.

## Electronic supplementary material

Supplementary Table 1, Supplementary Table 2

## Availability of data and materials

The data sets generated during and/or analysed during the current study are available for non-commercial use from the corresponding author on reasonable request.
