# Supplementary material for: Type 2 diabetes and risk of colorectal cancer in two large U.S. prospective cohorts
Source: Br J Cancer. 2018 Nov 7;119(11):1436–42. doi: 10.1038/s41416-018-0314-4 (PMC6265303; doi:10.1038/s41416-018-0314-4)
Supplement: Supplementary file 1 — Supplementary Table 1, Supplementary Table 2 [file 41416_2018_314_MOESM1_ESM.docx]

**Supplementary Table 1. Status and duration of type 2 diabetes and risk of subsites of colorectal cancer in the Nurses’ Health Study (NHS) and Health Professionals Follow-up Study (HPFS)**

|  | No diabetes | diabetes | 0.1–5 years | 5.1–10 years | 10.1–15 years | >15 years |
| --- | --- | --- | --- | --- | --- | --- |
| **Women (NHS)** |  |  |  |  |  |  |
| **Colon cancer (N=1746)** | **1266** | **129** | **40** | **32** | **22** | **35** |
| Age-adjusted model | **1** (Reference) | **1.35 (1.12, 1.62)** | 1.31 (0.96, 1.80) | 1.33 (0.93, 1.89) | 1.33 (0.87, 2.04) | **1.42 (1.01, 2.00)** |
| Multivariable-adjusted model* | **1** (Reference) | 1.18 (0.97, 1.44) | 1.14 (0.83, 1.58) | 1.14 (0.80, 1.64) | 1.16 (0.75, 1.78) | 1.29 (0.91, 1.83) |
| **Proximal colon cancer (N=1035)** | **754** | **80** | **28** | **19** | **12** | **21** |
| Age-adjusted model | **1** (Reference) | **1.32 (1.04, 1.66)** | **1.49 (1.02, 2.18)** | 1.23 (0.78, 1.95) | 1.14 (0.64, 2.02) | 1.30 (0.84, 2.02) |
| Multivariable-adjusted model* | **1** (Reference) | 1.20 (0.94, 1.54) | 1.34 (0.91, 1.98) | 1.10 (0.69, 1.75) | 1.04 (0.58, 1.87) | 1.24 (0.79, 1.95) |
| **Distal colon cancer (N=654)** | **474** | **44** | **12** | **11** | **8** | **13** |
| Age-adjusted model | 1 (Reference) | **1.37 (1.00, 1.88)** | 1.12 (0.63, 1.99) | 1.39 (0.76, 2.53) | 1.45 (0.72, 2.95) | 1.65 (0.94, 2.88) |
| Multivariable-adjusted model* | 1 (Reference) | 1.11 (0.80, 1.55) | 0.91 (0.51, 1.64) | 1.12 (0.60, 2.06) | 1.13 (0.55, 2.32) | 1.37 (0.77, 2.43) |
| **Rectal cancer (N=482)** | **365** | **30** | **11** | **10** | **2** | **7** |
| Age-adjusted model | 1 (Reference) | 1.20 (0.82, 1.75) | 1.34 (0.73, 2.45) | 1.57 (0.83, 2.96) | 0.46 (0.11, 1.84) | 1.16 (0.54, 2.46) |
| Multivariable-adjusted model* | 1 (Reference) | 1.11 (0.74, 1.65) | 1.23 (0.67, 2.28) | 1.41 (0.74, 2.71) | 0.42 (0.10, 1.69) | 1.10 (0.51, 2.37) |
| **Men (HPFS)** |  |  |  |  |  |  |
| **Colon cancer (N=983)** | **893** | **63** | **29** | **20** | **10** | **4** |
| Age-adjusted model | 1 (Reference) | **1.54 (1.18, 2.01)** | **1.48 (1.02, 2.17)** | **1.62 (1.03, 2.55)** | 1.54 (0.82, 2.92) | 1.62 (0.59, 4.41) |
| Multivariable-adjusted model* | 1 (Reference) | **1.40 (1.07, 1.84)** | 1.36 (0.93, 2.00) | 1.45 (0.92, 2.30) | 1.41 (0.74, 2.67) | 1.39 (0.51, 3.80) |
| **Proximal colon cancer (N=422)** | **380** | **30** | **17** | **8** | **3** | **2** |
| Age-adjusted model | 1 (Reference) | **1.62 (1.10, 2.37)** | **1.93 (1.17, 3.17)** | 1.38 (0.67, 2.81) | 1.02 (0.32, 3.21) | 1.95 (0.47, 8.09) |
| Multivariable-adjusted model* | 1 (Reference) | **1.56 (1.05, 2.32)** | **1.90 (1.14, 3.14)** | 1.30 (0.63, 2.68) | 0.98 (0.31, 3.12) | 1.79 (0.43, 7.52) |
| **Distal colon cancer (N=362)** | **341** | **14** | **6** | **5** | **2** | **1** |
| Age-adjusted model | 1 (Reference) | 1.08 (0.63, 1.87) | 0.90 (0.40, 2.04) | 1.34 (0.54, 3.30) | 1.06 (0.26, 4.37) | 1.60 (0.22, 11.80) |
| Multivariable-adjusted model* | 1 (Reference) | 0.89 (0.51, 1.55) | 0.76 (0.33, 1.73) | 1.10 (0.44, 2.73) | 0.82 (0.20, 3.41) | 1.19 (0.16, 8.91) |
| **Rectal cancer (N=265)** | **236** | **18** | **8** | **5** | **4** | **1** |
| Age-adjusted model | 1 (Reference) | **1.67 (1.02, 2.74)** | 1.62 (0.79, 3.33) | 1.46 (0.59, 3.59) | 2.31 (0.84, 6.38) | 1.47 (0.20, 11.00) |
| Multivariable-adjusted model* | 1 (Reference) | 1.48 (0.89, 2.47) | 1.46 (0.71, 3.04) | 1.28 (0.51, 3.18) | 2.03 (0.72, 5.69) | 1.33 (0.17, 10.10) |

*Adjusted for age, race (Whites or non-Whites), family history of diabetes (yes or no), regular aspirin use (yes or no), BMI (<25, 25-<27.5, 27.5-<30, ≥30 kg/m^2^), history of colorectal cancer in a parent or sibling (yes or no), history of endoscopy/sigmoidoscopy (yes or no), smoking (0, 0-<10, ≥10 pack-years), alcohol consumption (<5, 5-<15, ≥15 g/day), physical activity (<3, 3-<27, ≥27 METS-hours/week), total calorie (tertiles), energy-adjusted intake of total folate, total calcium, total vitamin D intake, processed meats, and beef, pork, or lamb as a main dish (all in tertiles). Women were also adjusted for postmenopausal hormone use (premenopausal, never, past, or current user).

**Supplementary Table 2. Status and duration of untreated* type 2 diabetes and colorectal cancer risk in the Nurses’ Health Study (NHS) and Health Professionals Follow-up Study (HPFS)**

|  | **Women (NHS, N=1644)** | | **Men (HPFS, N=1161)** | |
| --- | --- | --- | --- | --- |
|  | Cases | HR (95% CI) | Cases | HR (95% CI) |
| **No diabetes** | 1570 | 1 (Reference) | 1118 | 1 (Reference) |
| **Diabetes** |  |  |  |  |
| Age-adjusted model | 74 | 1.16 (0.92, 1.47) | 43 | **1.46 (1.07, 2.00)** |
| Multivariable-adjusted model** | 74 | 1.02 (0.80, 1.31) | 43 | **1.38 (1.01, 1.90)** |
| **0.1–5 years** |  |  |  |  |
| Age-adjusted model | 15 | 1.03 (0.62, 1.72) | 22 | 1.36 (0.88, 2.09) |
| Multivariable-adjusted model** | 15 | 0.89 (0.53, 1.50) | 22 | 1.28 (0.83, 1.98) |
| **5.1–10 years** |  |  |  |  |
| Age-adjusted model | 16 | 1.21 (0.73, 1.98) | 16 | **1.84 (1.11, 3.05)** |
| Multivariable-adjusted model** | 16 | 1.03 (0.63, 1.71) | 16 | **1.72 (1.04, 2.86)** |
| **10.1–15 years** |  |  |  |  |
| Age-adjusted model | 11 | 0.97 (0.53, 1.76) | 4 | 1.27 (0.47, 3.44) |
| Multivariable-adjusted model** | 11 | 0.86 (0.47, 1.56) | 4 | 1.21 (0.45, 3.29) |
| **>15 years** |  |  |  |  |
| Age-adjusted model | 32 | 1.30 (0.91, 1.84) | 1 | 0.76 (0.11, 5.49) |
| Multivariable-adjusted model** | 32 | 1.18 (0.82, 1.69) | 1 | 0.75 (0.10, 5.44) |

*Individuals with type 2 diabetes who have used hypoglycemic medication (insulin or hypoglycemic agent) were excluded. **Adjusted for age, race (Whites or non-Whites), family history of diabetes (yes or no), regular aspirin use (yes or no), BMI (<25, 25-<27.5, 27.5-<30, ≥30 kg/m^2^), history of colorectal cancer in a parent or sibling (yes or no), history of endoscopy/sigmoidoscopy (yes or no), smoking (0, 0-<10, ≥10 pack-years), alcohol consumption <5, 5-<15, ≥15 g/day), physical activity (<3, 3-<27, ≥27 METS-hours/week), total calorie (tertiles), energy-adjusted intake of total folate, total calcium, total vitamin D intake, processed meats, and beef, pork, or lamb as a main dish (all in tertiles). We also adjusted for postmenopausal hormone use (premenopausal, never, past, or current user) in women.
